# Supplementary material for: Design and Evaluation of Short Bovine Lactoferrin-Derived Antimicrobial Peptides against Multidrug-Resistant Enterococcus faecium
Source: Antibiotics (Basel). 2022 Aug 10;11(8):1085. doi: 10.3390/antibiotics11081085 (PMC9404989; doi:10.3390/antibiotics11081085)
Supplement: Supplementary file 1 [file antibiotics-11-01085-s001.zip › antibiotics-1850968-supplementary.pdf]

Supplementary materials

Design and Evaluation of Short Bovine Lactoferrin-Derived Antimicrobial Peptides against Multidrug-Resistant *Enterococcus faecium*

Biswajit Mishra [1,*], LewisOscar Felix [1], Anindya Basu [2,3], Sai Sundeep Kollala [4], Yashpal Singh Chhonker [4], Narchonai Ganesan [1], Daryl J. Murry [4] and Eleftherios Mylonakis [1,*]

[1] Infectious Diseases Division, Warren Alpert Medical School of Brown University, Providence, RI 02903, USA; lewis_oscar_felix_raj_lucas@brown.edu (L.F.); narchonai_ganesan@brown.edu (N.G.)
[2] School of Pharmaceutical Sciences, Rajiv Gandhi Technological University, Bhopal 462033, India; anindya@rgtu.net
[3] School of Biotechnology, Rajiv Gandhi Technological University, Bhopal 462033, India
[4] Department of Pharmacy Practice and Science, College of Pharmacy, University of Nebraska Medical Center, Omaha, NE 68198, USA; saisundeep.kollala@unmc.edu (S.S.K.); y.chhonker@unmc.edu (Y.S.C.); dj.murry@unmc.edu (D.J.M.)
* Correspondence: biswajit_mishra@brown.edu (B.M.); emylonakis@lifespan.org (E.M.)

Contents:

Table S1: Bacterial strain used in this study.

Table S2: Primers used in this study for biofilm genes through real time PCR.

Table S3: MIC (µg/ml) of LfcinB6 designed peptides against *S. aureus* and *E. faecalis*.

Table S4: Hydrogen bonds between peptide 5L with nearest POPC and cardiolipin molecules.

Table S5: Significantly altered ($p < 0.05$) 13 primary metabolites in 5L treated groups comparted to control *E. faecium* strain 68.

Table S6: Important features selected by fold-change analysis with threshold 2 of primary metabolites in 5L treated groups comparted to control *E. faecium* strain 68.

Figure S1: Disruption of 24 h established biofilms of *E. faecium* strain E007 (a tetracycline-resistant strain) by peptides 5L and 6L. (A) live-cell reductions by XTT, and (B) biomass by CV.

Figure S2: Partial density plot of the MD simulation representing peptide 5L (red) remained predominantly in about 1.25 nm inside the upper membrane leaflet (black).

Figure S3: (A) Principal component analysis (PCA) and (B) partial least squares-discriminant analysis (PLSDA) plots of metabolites between *E. faecium* control and 5L treated conditions with three technical replicates.

Figure S4: AlphaFold2 derived structures of LfcinB6 derived peptides and helical wheel representations.

Figure S5: Enrichment analysis of metabolic pathways derived from peptide 5L treated E. faecium.

Figure S6: The MS and HPLC information of synthetic peptides.

References

Table S1. Bacterial strain used in this study.

Bacterial strains	Resistance	References
E. faecium		
C68	Ampicillin and vancomycin resistant	[1]
D14	Ampicillin resistant	[2]
D24	Ampicillin resistant	[2]
D25	Ampicillin resistant	[2]
D29	Ampicillin resistant	[2]
WC176	Ampicillin and vancomycin resistant	[3]
E007	Tetracycline resistant	[4]
E. faecalis		
V583	vancomycin resistant	[5]
OG1RF	rifampin and fusidic acid resistant	[6]
MMH594	multidrug-resistant	[7]
***S. aureus* MW2**	MRSA	[8]

Table S2. Primers used in this study for biofilm genes through real time PCR.

Name of the genes	Oligonucleotide sequences (5'-3')	Lengths (bp)	References
esp	F: CGGTCATACCGACGACCAAA<br>R: TGTCACATCGCCATCGACTT	745	[9]
ace	F: CAAGCATTATTGGCAGCGTT<br>R: TCTATCACATTCGGTTGCG	320	[10]
ebpA	F: CCATTTGCAGAAGCAAGAATG<br>R: GAGTGAAAGTTCCTCCTCTAG	613	[11]
ebpC	F: CTGCTACGAATATGGTGGTG<br>R: GGTGTTTGATTGTTTGCTTC	487	[11]
16S rDNA	F: AGAGTTTGATCMTGGCTCAG<br>R: TACGGYTACCTTGTTACGACTT	1506	[12]

Table S3. Minimal inhibitory concentration (µg/ml) of designed peptides against *S. aureus* and *E. faecalis*.

**Peptide**	**MIC (µg/ml)**			
	S. aureus **MW2**	***E. faecalis*** **V583**[a]	***E. faecalis*** **OG1RF**[b]	***E. faecalis*** **MMH594**[c]
1L	> 32	> 32	> 32	> 32
2L	> 32	> 32	> 32	> 32
3L	> 32	> 32	> 32	> 32
4L	> 32	> 32	> 32	> 32
5L	> 32	> 32	> 32	> 32
6L	32	> 32	> 32	> 32
7L	> 32	> 32	> 32	> 32
8L	> 32	> 32	> 32	> 32
Vancomycin	1	> 32	> 32	> 32

Table S4: Hydrogen bonds between peptide 5L with nearest POPC and cardiolipin molecules.

DONOR	ACCEPTOR	OCCUPANCY
CHL19-Side	ARG1-Main	5.88%
CHL12-Side	LEU10-Main	75.49%
TRP5-Side	CHL14-Side	1.96%
TRP3-Side	CHL14-Side	0.98%
TRP11-Side	CHL12-Side	2.94%
TRP5-Side	CHL11-Side	0.98%
CHL11-Side	TRP9-Side	0.98%
TRP3-Side	CHL19-Side	0.98%

Table S5: Significantly altered ($p < 0.05$) 13 primary metabolites in 5L treated groups comparted to control *E. faecium* strain 68.

Sl. No.	Compounds	t.stat	p.value	-log10(p)
1	Succinic acid	-4.7196	0.009	2.037
2	Tryptophan	-4.2563	0.013	1.883
3	Putriscine	3.6004	0.022	1.643
4	Methionine	-3.6003	0.022	1.643
5	Cysteine	-3.5138	0.024	1.609
6	Isocitric acid	-3.2734	0.030	1.513
7	Guanine	-3.0727	0.037	1.429
8	4-Aminobutryc acid	3.0604	0.037	1.424
9	Citicoline	-3.0046	0.039	1.400
10	Citric acid	-2.8826	0.044	1.347
11	Malic acid	-2.8521	0.047	1.322
12	Oxidized glutathione	-2.8078	0.048	1.314
13	Arginosuccinic acid	-2.7301	0.052	1.280
14	Fumaric acid	-2.6724	0.055	1.254
15	Creatinine	2.6672	0.055	1.252
16	Cystathionine	-2.6622	0.056	1.249
17	Threonine	-2.6493	0.057	1.244
18	Creatinine.1	2.648	0.057	1.243
19	Histidine	-2.4687	0.069	1.160
20	Aspartic acid	-2.4629	0.069	1.158
21	Alanine	-2.3502	0.078	1.105
22	Guanosine	-2.3399	0.079	1.100
23	Adenylsuccinic acid	-2.2861	0.084	1.074
24	Xanthosine	-2.2613	0.086	1.062
25	Isoleucine	-2.2284	0.089	1.046
26	Phenylalanine	-2.225	0.090	1.045
27	Valine	-2.2079	0.091	1.037
28	Glutamic acid	-2.1742	0.095	1.020
29	Dopa	-2.1691	0.095	1.018

Table S6: Important features selected by fold-change analysis with threshold 2 of primary metabolites in 5L treated groups comparted to control *E. faecium* strain 68.

Sl. No.	Compounds	Fold change	Log2 (FC)
1	Serine	0.033	-4.88
2	Cysteine	0.047	-4.40
3	Guanine	0.130	-2.94
4	Putriscine	7.197	-2.84
5	Methionine	0.175	-2.50
6	Tryptophan	0.178	-2.48
7	Aspartic acid	0.197	-2.34
8	Succinic acid	0.204	-2.29
9	Arginosuccinic acid	0.204	-2.29
10	Adenylsuccinic acid	0.226	-2.14
11	Uridine	0.228	-2.12
12	4-Aminobutryc acid	4.319	2.11
13	Citicoline	0.233	-2.10
14	Isocitric acid	0.237	-2.07
15	Inosine	0.239	-2.06
16	Citric acid	0.244	-2.03
17	S-Adenosylhomocysteine	0.267	-1.90
18	Guanosine	0.275	-1.85
19	Adenine	0.311	-1.68
20	Xanthosine	0.315	-1.66
21	Thymidine	0.320	-1.64
22	Dopamine	3.107	1.63
23	Dopa	0.337	-1.56
24	Serotonin	2.886	1.52
25	Cystathionine	0.369	-1.43
26	Leucine	0.373	-1.42
27	Allantoin	2.67	1.42
28	Glutathione	0.376	-1.40
29	Ornitine	0.378	-1.40
30	Alanine	0.383	-1.38
31	Threonine	0.384	-1.38
32	Fumaric acid	0.387	-1.36
33	Malic acid	0.392	-1.35
34	Oxidized glutathione	0.403	-1.30
35	NAD	0.415	-1.26
36	Histidine	0.420	-1.25
37	Phenylalanine	0.423	-1.23
38	Symmetric dimethylarginine	2.347	1.23
39	Valine	0.433	-1.20
40	Cobalamine	2.285	1.19
41	Carbamoyl aspertate	2.212	1.14
42	Isoleucine	0.458	-1.12
43	Kynureine	2.152	1.10
44	2-Aminobutryc acid	0.480	-1.05

Figure S1: Disruption of 24 h established biofilms of *E. faecium* strain E007 (a tetracycline-resistant strain) by peptides 5L and 6L. (A) live-cell reductions by XTT, and (B) biomass by CV.

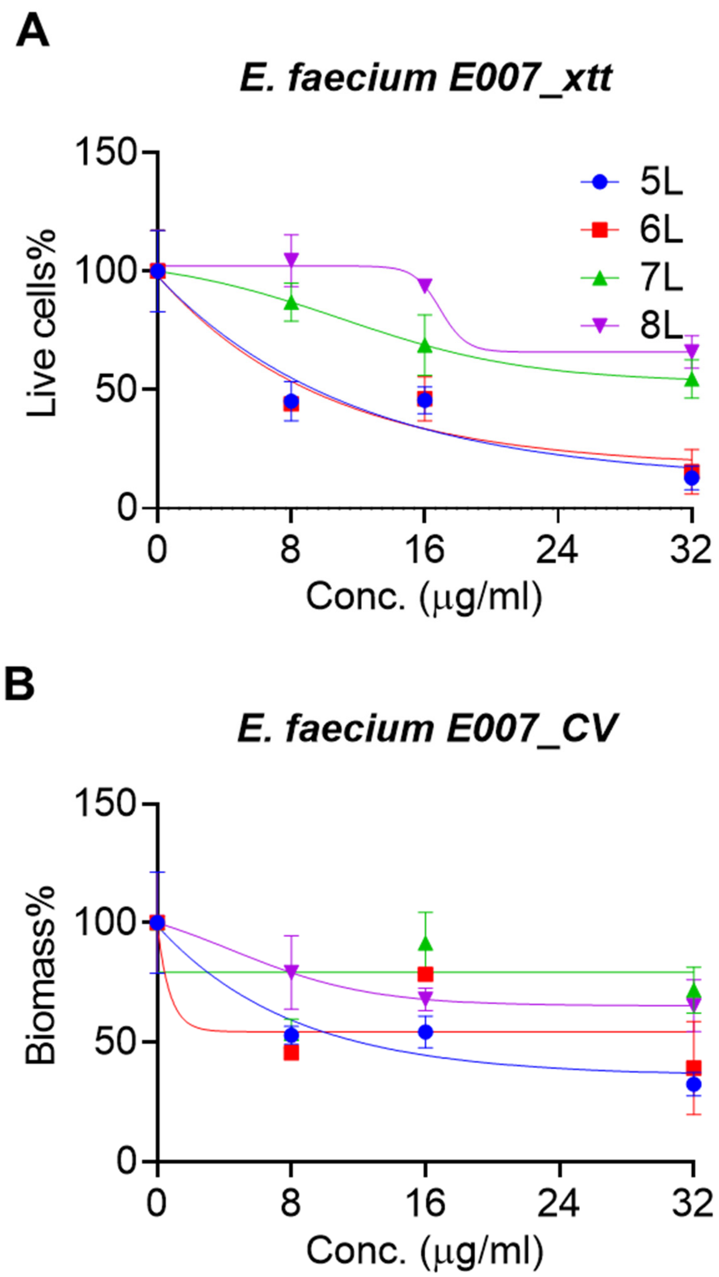

Figure S2: Partial density plot of the MD simulation representing peptide 5L (red) remained predominantly in about 1.25 nm inside the upper membrane leaflet (black).

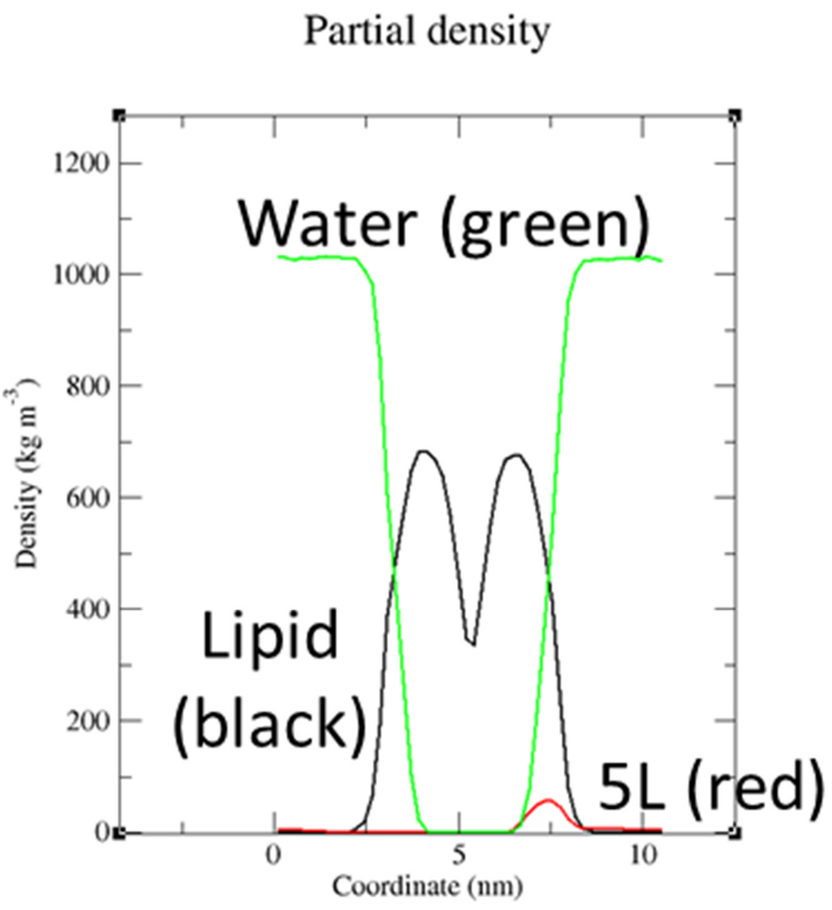

Figure S3: (A) Principal component analysis (PCA) and (B) partial least squares-discriminant analysis (PLSDA) plots of metabolites between *E. faecium* control and 5L treated conditions with three technical replicates.

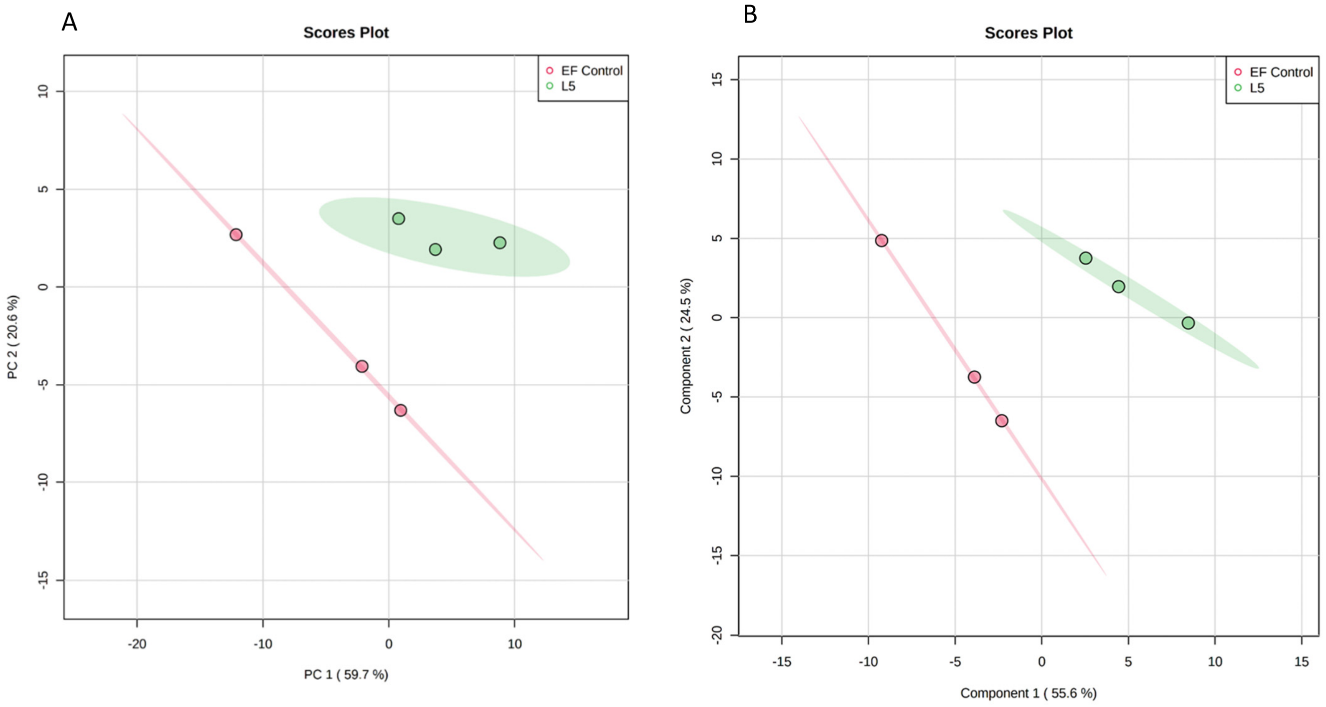

Both graphs show that the *E. faecium* control (red) and 5L treated (green) bacterial samples are separated from the other groups, indicating that a very distinct metabolite composition. Component 1 indicates the degree of variation between the groups based on their total metabolite content, and component 2 indicates the differences within the groups.

Figure S4: AlphaFold2 derived structures of LfcinB6 derived peptides. Surface representation of peptides with red as charged amino acids and gray as hydrophobic amino acids) and helical wheel representations.

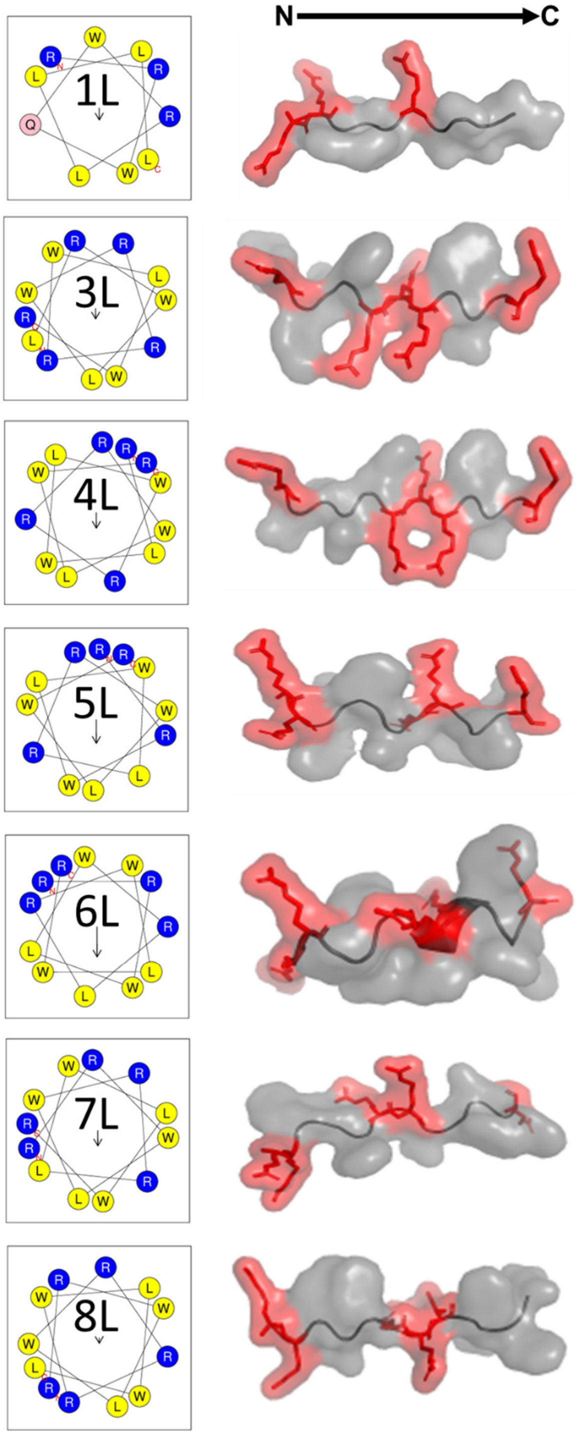

Figure S5: Enrichment analysis of metabolic pathways derived from peptide 5L treated *E. faecium*.

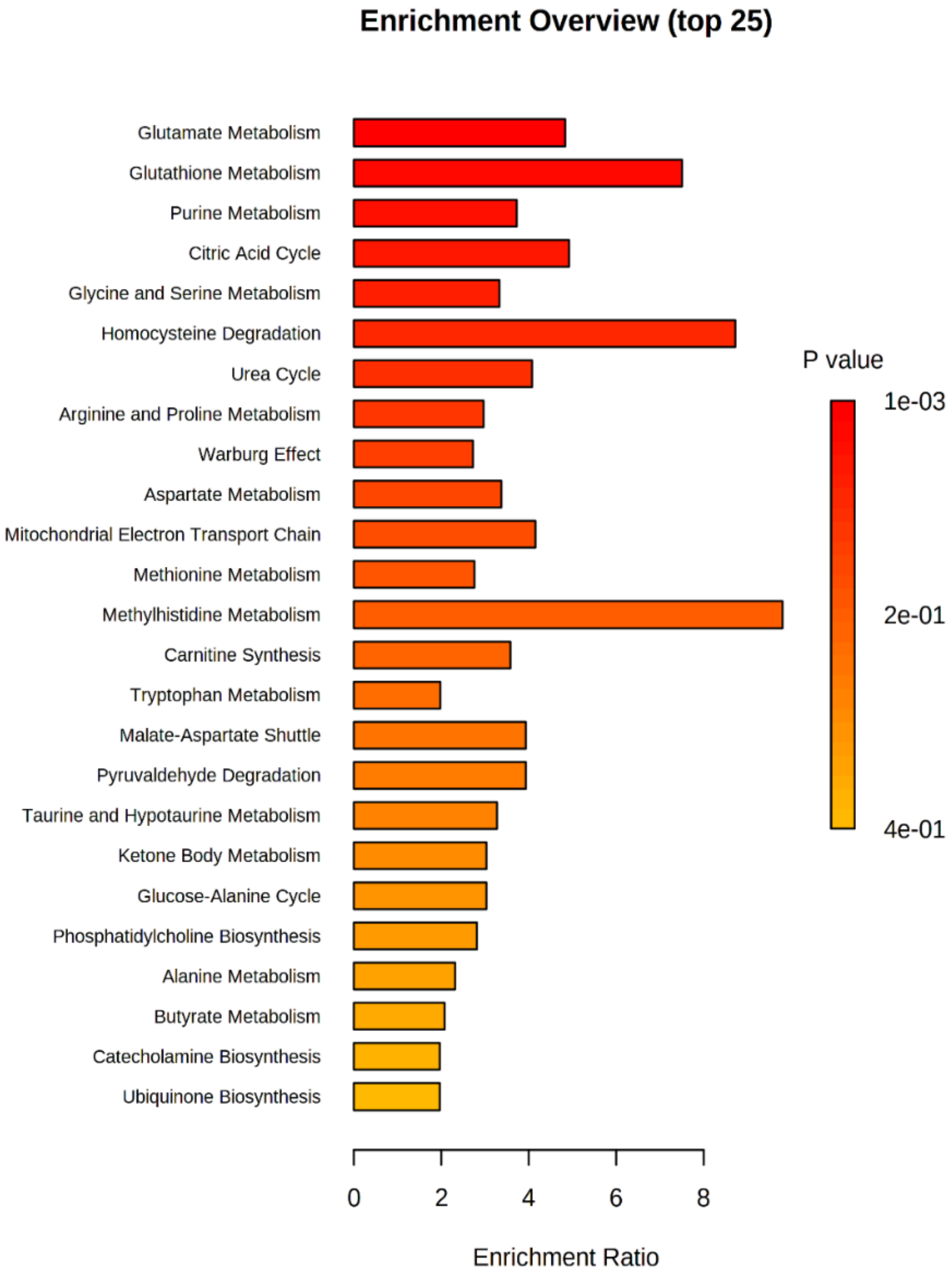

Figure S6: The MS and HPLC information of synthetic peptides.

Sample Name :1L
Sample ID :U929RHA240-1
Time Processed :13:34:59
Month-Day-Year Processed :02/04/2022

Pump A : 0.065% trifluoroacetic in 100% water (v/v)
Pump B : 0.05% trifluoroacetic in 100% acetonitrile (v/v)
Total Flow:1 ml/min
Wavelength:220 nm
<<LC Time Program>>

Time	Module	Command	Value
0.01	Pumps	B.Conc	5
25.00	Pumps	B.Conc	65
25.01	Pumps	B.Conc	95
27.00	Pumps	B.Conc	95
27.01	Pumps	B.Conc	5
35.00	Pumps	B.Conc	5
35.01	Controller	Stop	

<<Column Performance>>
<Detector A>
Column :Inertsil ODS-3 4.6 x 250 mm
Equipment: ZJ20010140

<Chromatogram>

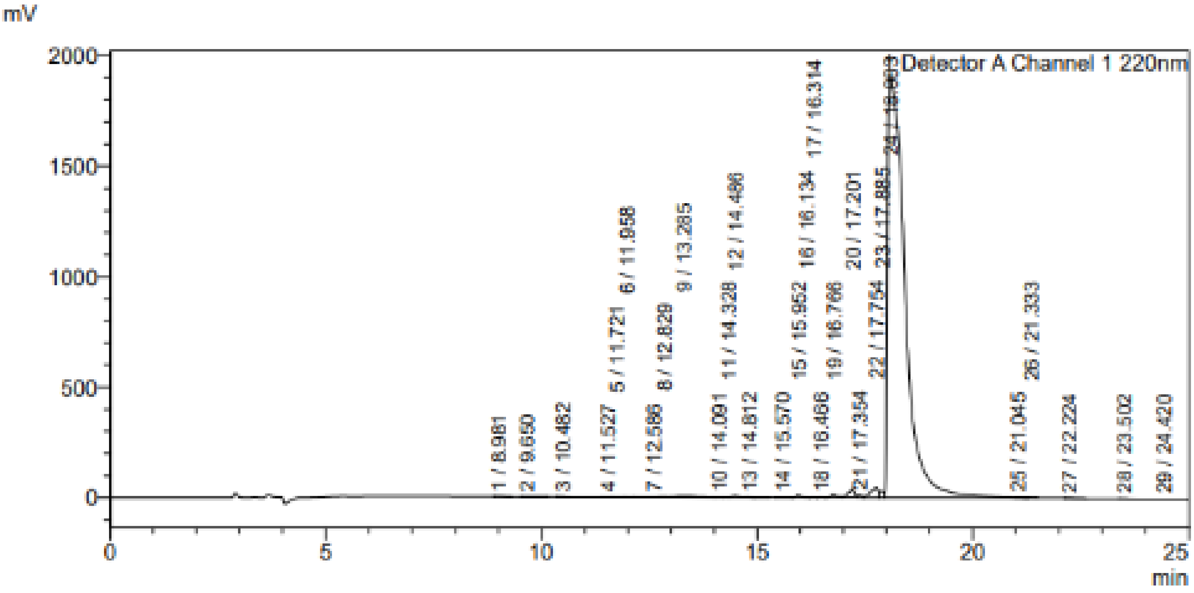

Mass Spectrum

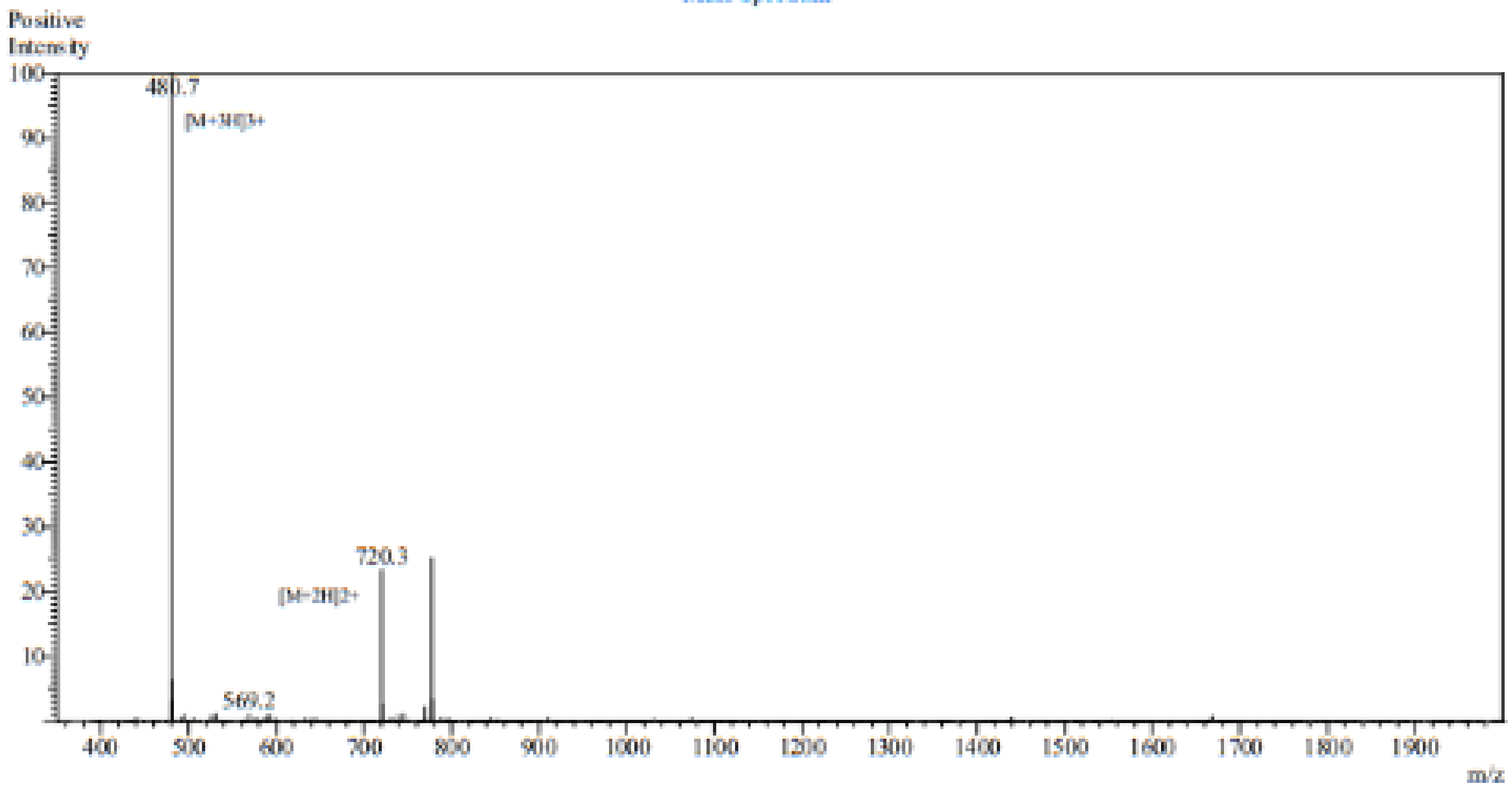

Sample Information	
Month-Day Processed :	02/02/22
Time Processed :	21:32:06
Injection Volume :	0.4
Sample Name :	1L
Sample ID :	U0298HA240-1
Theoretical MW :	1438.77
Observed MW :	1439.1

Interface	: ESI
Nebulizing Gas Flow	: 1.5L/min
CDL Temp	: 250
Block Temp	: 200

Equipment	: GK11010007
Interface Bias	: +4.5 kV
Drying Gas Flow	: 5 L/min
T.Flow	: 0.2 ml/min
B.conc	: 50%H2O/50%MeOH

Sample Name :2L
Sample ID :U929RHA240-3
Time Processed :6:03:38 PM
Month-Day-Year Processed :02/03/2022

Pump A : 0.065% trifluoroacetic in 100% water (v/v)
Pump B : 0.05% trifluoroacetic in 100% acetonitrile (v/v)
Total Flow:1 ml/min
Wavelength:220 nm

Time	Module	Command	Value
0.01	Pumps	B.Conc	5
25.00	Pumps	B.Conc	65
25.01	Pumps	B.Conc	95
27.00	Pumps	B.Conc	95
27.01	Pumps	B.Conc	5
35.00	Pumps	B.Conc	5
35.01	Controller	Stop	

<<Column Performance>>
<Detector A>
Column : Inertsil ODS-3 4.6 x 250 mm

Chromatogram

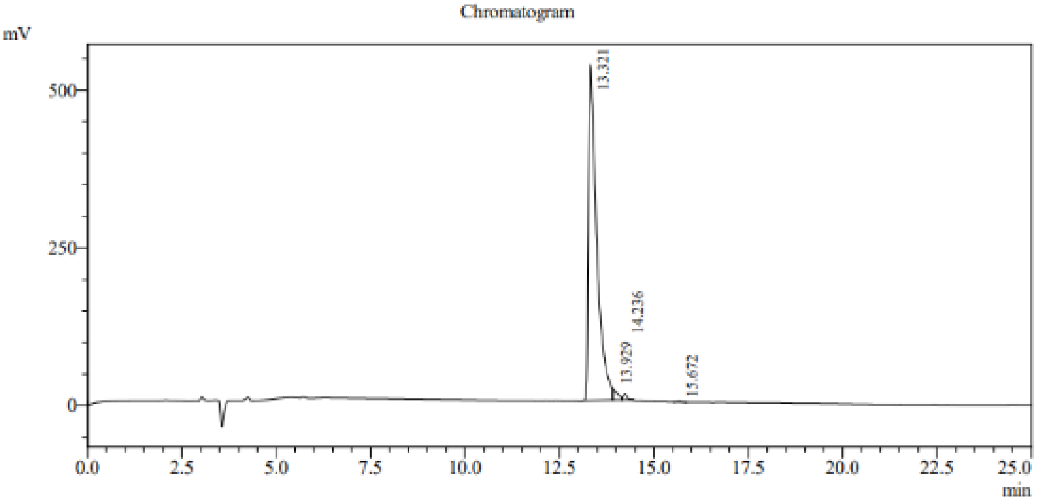

1 Detector A Channel 1 / 220nm

Mass Spectrum

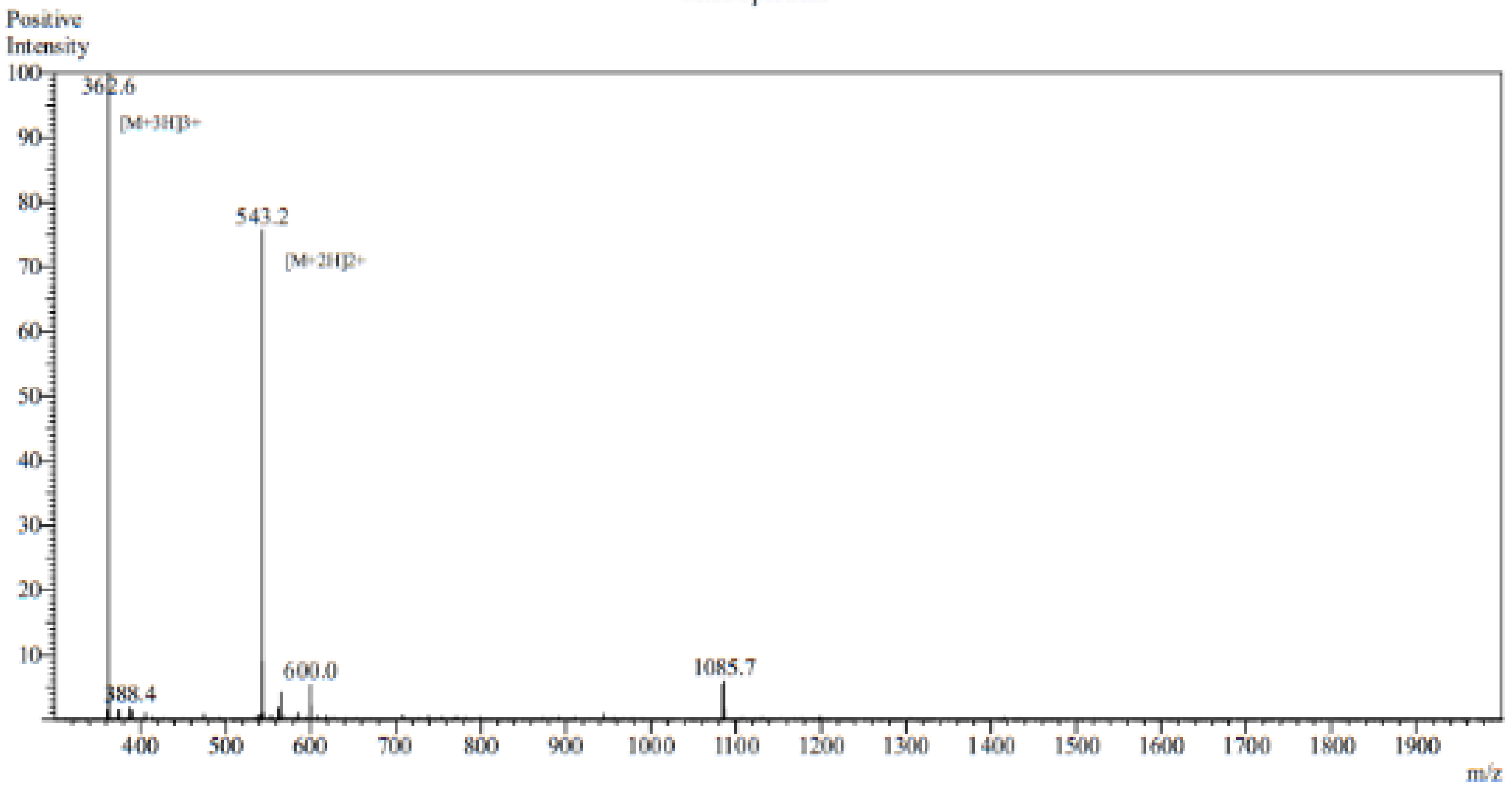

Sample Information
Month-Day Processed : 02/03/22
Time Processed : 20:29:53
Injection Volume : 0.5
Sample Name : 2L
Sample ID : U929RHA240-3
Theoretical MW : 1084.33
Observed MW : 1084.8

Interface :ESI
Nebulizing Gas Flow : 1.5L/min
CDL Temp :250
Block Temp :200

Equipment : ZJ21010035
Interface Bias : +4.5 kV
Drying Gas Flow : 5 L/min
T.Flow :0.2 ml/min
Bcone :50%H2O/50%MeOH

Sample Name : 3L
Sample ID : U929RHA240-5
Time Processed : 15:44:06
Month-Day-Year Processed : 02/12/2022

Pump A : 0.065% trifluoroacetic in 100% water (v/v)
Pump B : 0.05% trifluoroacetic in 100% acetonitrile (v/v)
Total Flow:1 ml/min
Wavelength:220 nm

Time	Module	Command	Value
0.01	Pumps	Pump A B.Conc	5
25.00	Pumps	Pump A B.Conc	65
25.01	Pumps	Pump A B.Conc	95
27.00	Pumps	Pump A B.Conc	95
27.01	Pumps	Pump A B.Conc	5
35.00	Pumps	Pump A B.Conc	5
35.01	Controller	Stop	

<<Column Performance>>
<Detector A>
Column : Inertsil ODS-3 4.6 x 250 mm
Equipment: GK11010017

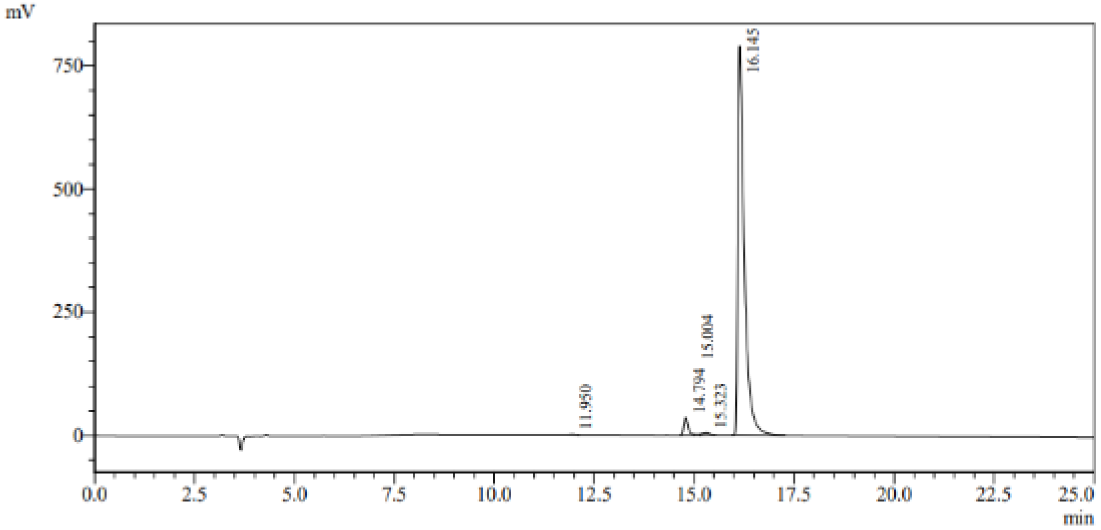

1 Detector A Channel 1 / 220nm

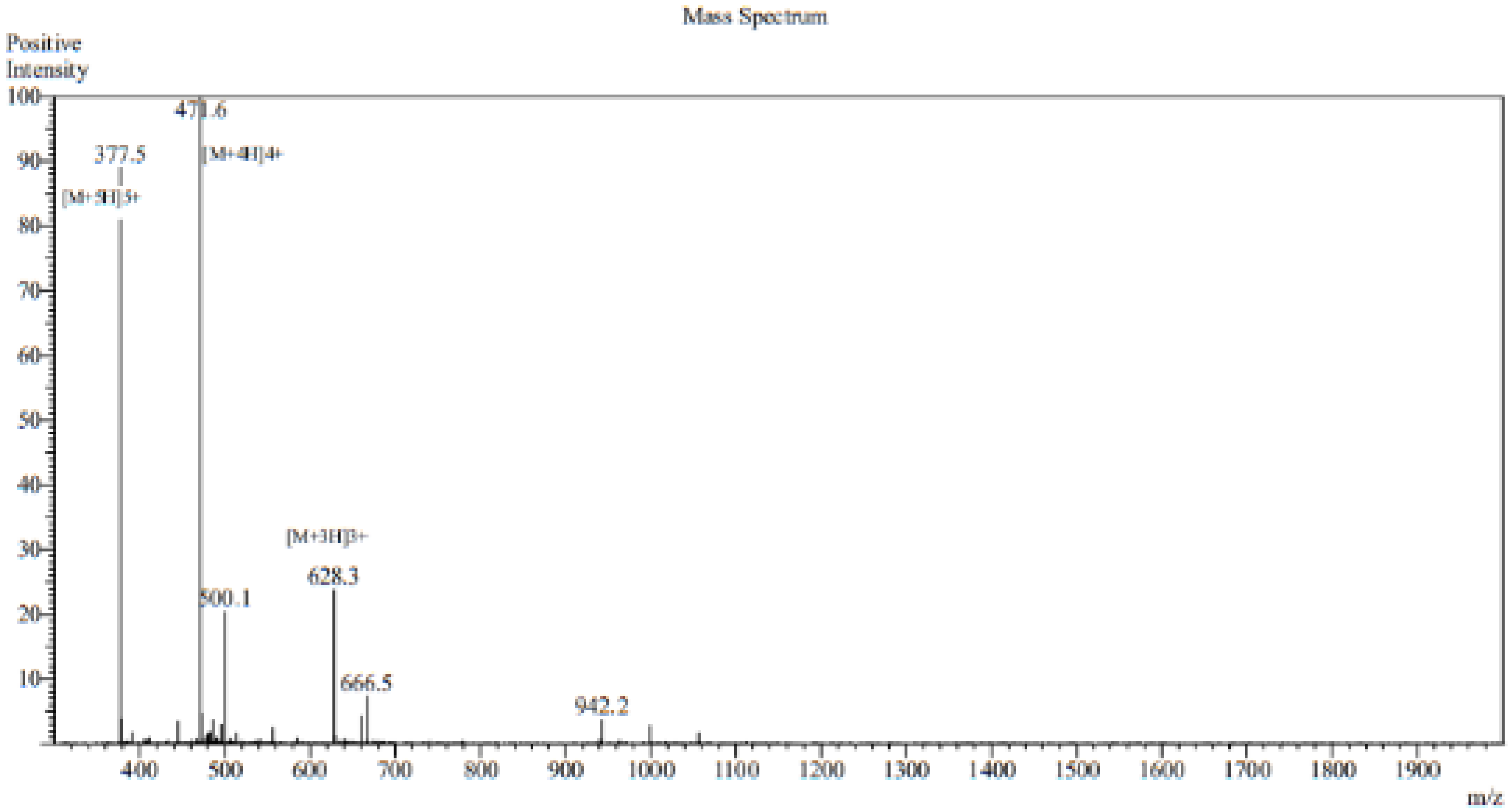

Sample Information
Month-Day Processed : 02/11/22
Time Processed : 3:01:41 PM
Injection Volume : 0.1
Sample Name : 3L
Sample ID : U929RHA240-5
Theoretical MW : 1882.28
Observed MW : 1882.4

Interface :ESI
Nebulizing Gas Flow :1.5L/min
CDL Temp :250
Block Temp :200

Equipment : GK11010007
Interface Bias : +4.5 kV
Drying Gas Flow :5 L/min
T.Flow :0.2 ml/min
B.conc :50%H2O/50%MeOH

Sample Name :4L
Sample ID :U929RHA240-7
Time Processed :4:14:58
Month-Day-Year Processed :02/15/2022

Pump A : 0.065% trifluoroacetic in 100% water (v/v)
Pump B : 0.05% trifluoroacetic in 100% acetonitrile (v/v)
Total Flow:1 ml/min
Wavelength:220 nm

Time	Module	Command	Value
0.01	Pumps	B.Conc	5
25.00	Pumps	B.Conc	65
25.01	Pumps	B.Conc	95
27.00	Pumps	B.Conc	95
27.01	Pumps	B.Conc	5
35.00	Pumps	B.Conc	5
35.01	Controller	Stop	

<<Column Performance>>
<Detector A>
Column : Inertsil ODS-3 4.6 x 250 mm

Chromatogram

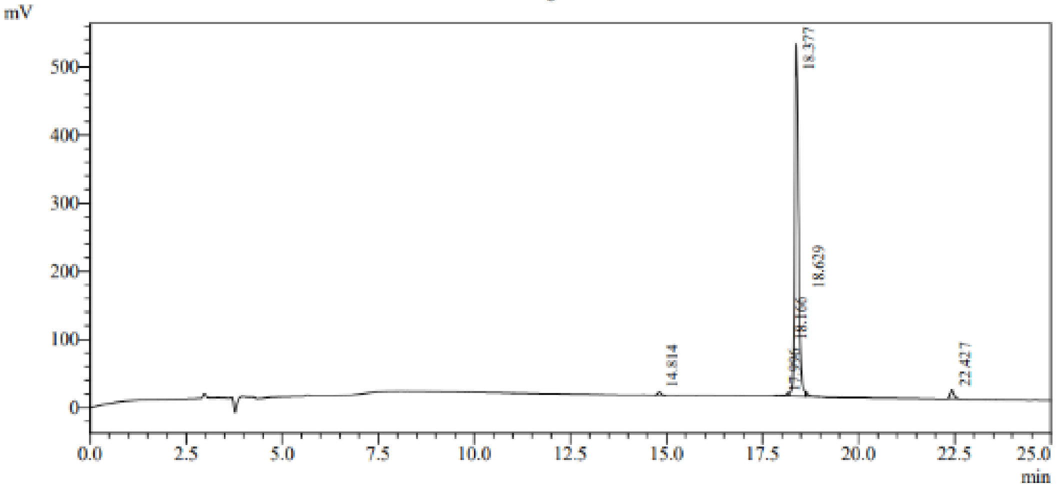

1 Detector A Channel 1 / 220nm

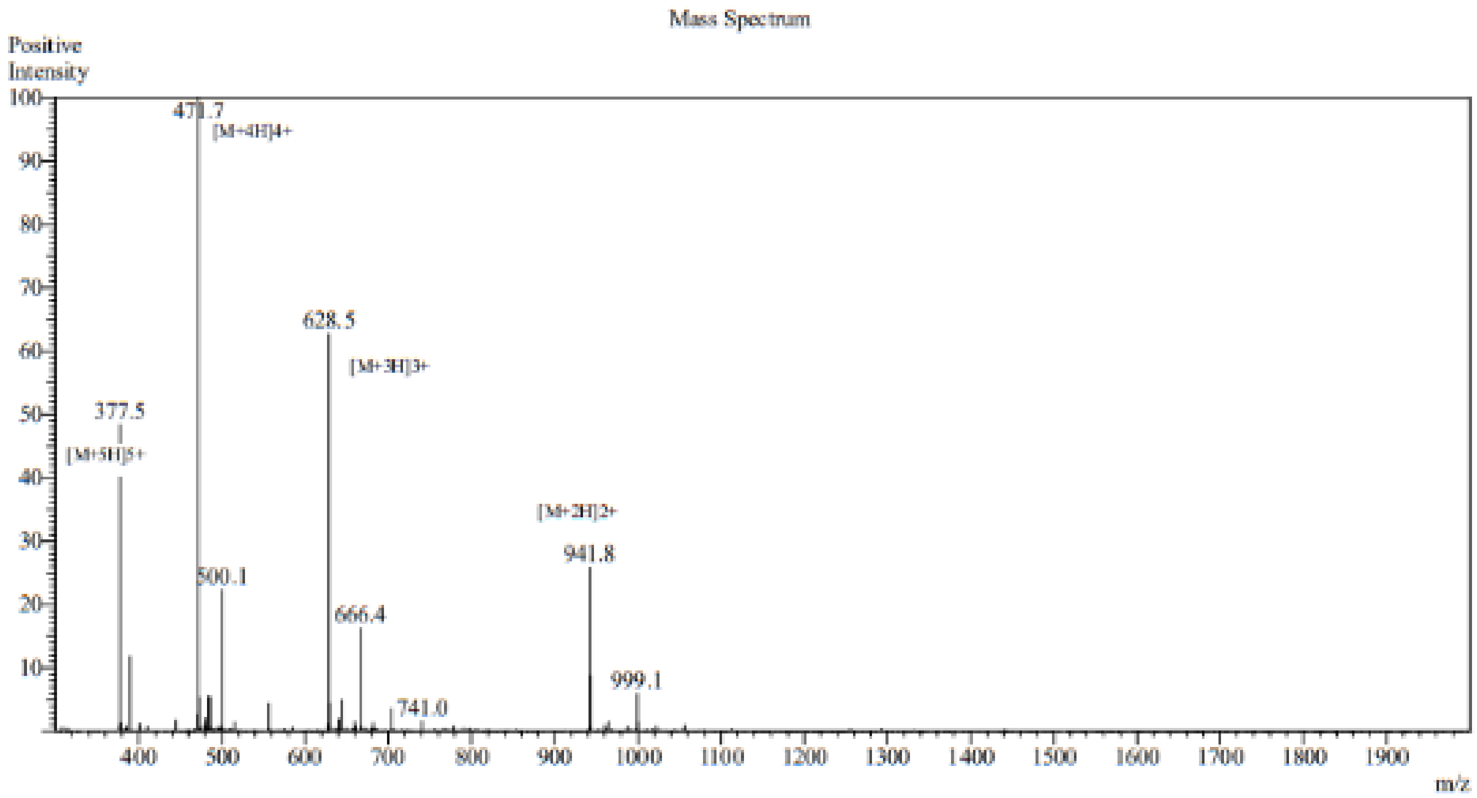

Sample Information
Month-Day Processed : 02/15/22
Time Processed : 15:04:11
Injection Volume : 0.4
Sample Name : 4L
Sample ID : U929RHA240-7
Theoretical MW : 1882.28
Observed MW : 1882.8

Interface :ESI
Nebulizing Gas Flow :1.5L/min
CDL Temp :250
Block Temp :200

Equipment : ZJ21010035
Interface Bias : +4.5 kV
Drying Gas Flow :5 L/min
T.Flow :0.2 ml/min
B.conc :50%H2O/50%MeOH

Sample Name : 5L
Sample ID : U929RHA240-9
Time Processed : 17:15:10
Month-Day-Year Processed : 02/15/2022

Pump A : 0.065% trifluoroacetic in 100% water (v/v)
Pump B : 0.05% trifluoroacetic in 100% acetonitrile (v/v)
Total Flow: 1 ml/min
Wavelength: 220 nm

Time	Module	Command	Value
0.01	Pumps	B.Conc	5
25.00	Pumps	B.Conc	65
25.01	Pumps	B.Conc	95
27.00	Pumps	B.Conc	95
27.01	Pumps	B.Conc	5
35.00	Pumps	B.Conc	5
35.01	Controller	Stop	

<<Column Performance>>
<Detector A>
Column : Inertsil ODS-3 4.6 x 250 mm
Equipment: GK11010017

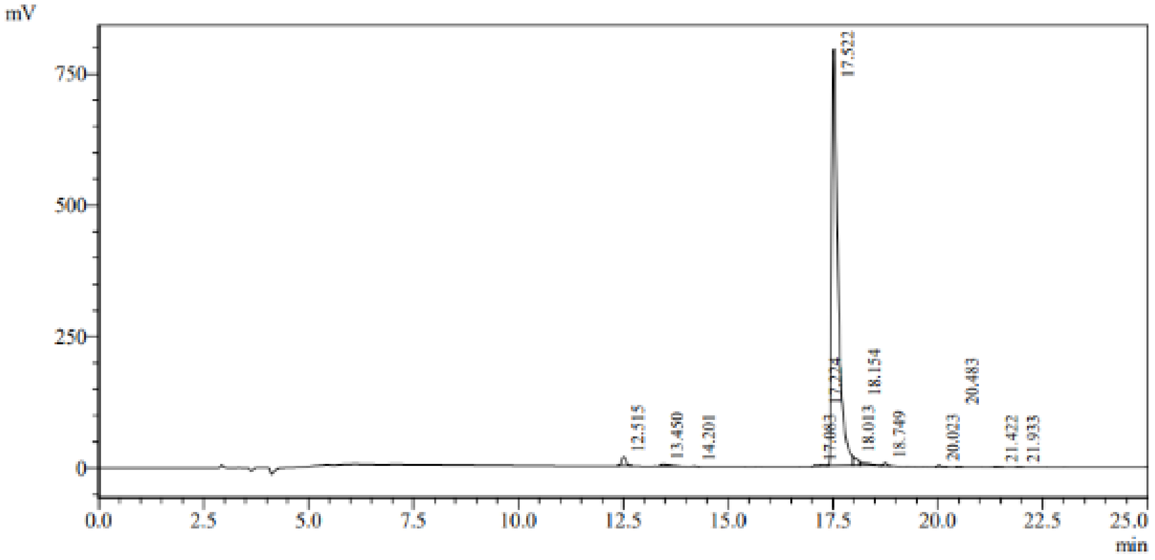

1 Detector A Channel 1 / 220nm

Mass Spectrum

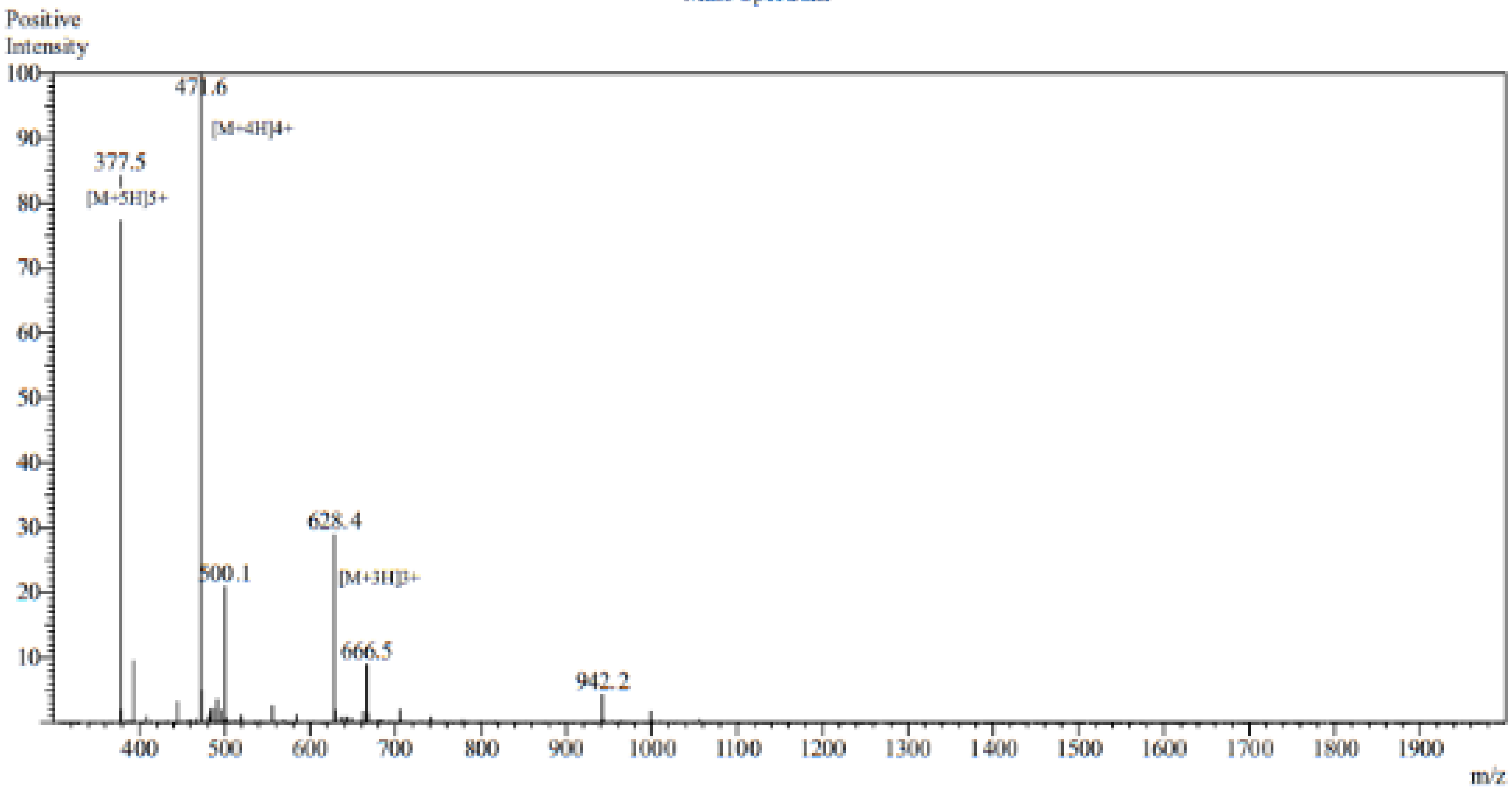

Sample Information	
Month-Day Processed :	02/15/22
Time Processed :	4:29:13 PM
Injection Volume :	0.2
Sample Name :	5L
Sample ID :	U929RHA240-9
Theoretical MW :	1882.28
Observed MW :	1882.4

Interface	:ESI
Nebulizing Gas Flow	:1.5L/min
CDL Temp	:250
Block Temp	:200

Equipment	: ZJ21010035
Interface Bias	: +4.5 kV
Drying Gas Flow	:5 L/min
T.Flow	:0.2 ml/min
B.conc	:50%H2O/50%MeOH

Sample Name :6L
Sample ID :U929RHA240-11
Time Processed :5:32:20 AM
Month-Day-Year Processed :02/03/2022

Pump A : 0.065% trifluoroacetic in 100% water (v/v)
Pump B : 0.05% trifluoroacetic in 100% acetonitrile (v/v)
Total Flow:1 ml/min
Wavelength:220 nm

Time	Module	Command	Value
0.01	Pumps	Pump A B.Conc	5
25.00	Pumps	Pump A B.Conc	65
25.01	Pumps	Pump A B.Conc	95
27.00	Pumps	Pump A B.Conc	95
27.01	Pumps	Pump A B.Conc	5
35.00	Pumps	Pump A B.Conc	5
35.01	Controller	Stop	

<<Column Performance>>
<Detector A>
Column : Inertsil ODS-3 4.6 x 250 mm

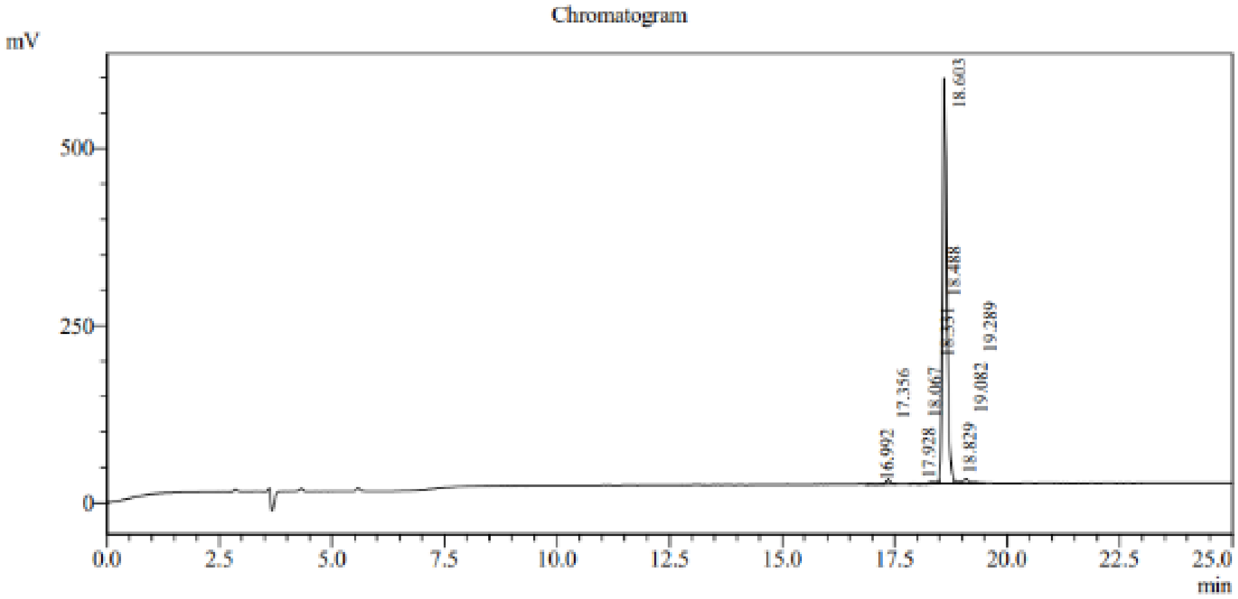

1 Detector A Channel 1 / 220nm

Mass Spectrum

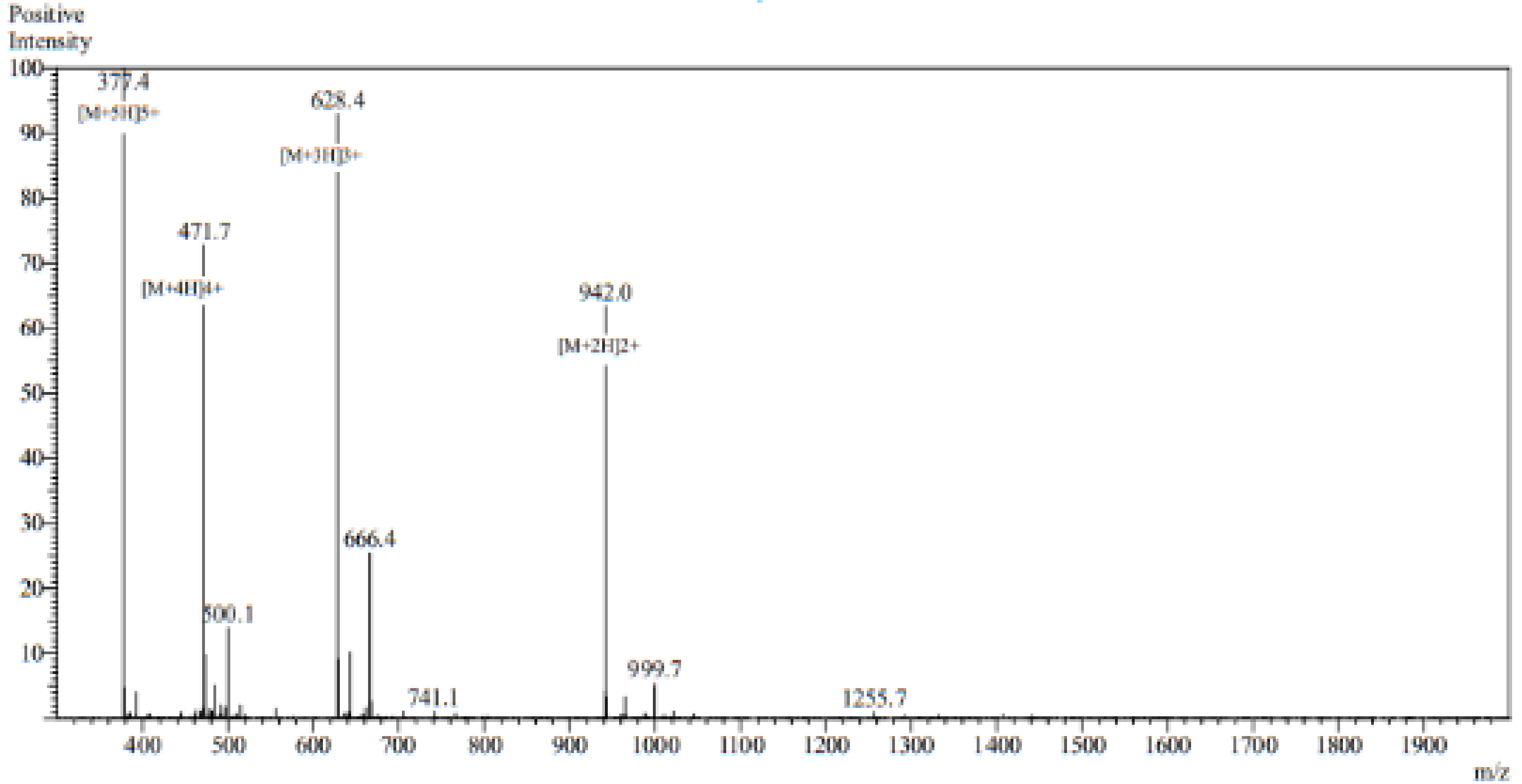

Sample Information	
Month Day Processed :	02/03/22
Time Processed :	13:23:08
Injection Volume :	0.4
Sample Name :	6L
Sample ID :	U929RHA240-11
Theoretical MW :	1882.28
Observed MW :	1882.0

Interface	:ESI
Nebulizing Gas Flow	:1.5L/min
CDL Temp	:250
Block Temp	:200

Equipment	: ZJ21010035
Interface Bias	: +4.5 kV
Drying Gas Flow	: 5 L/min
T.Flow	:0.2 ml/min
B.conc	:50%H2O/50%MeOH

Sample Name : 7L
Sample ID : U929RHA240-13
Time Processed : 11:07:40
Month-Day-Year Processed : 02/12/2022

Pump A : 0.065% trifluoroacetic in 100% water (v/v)
Pump B : 0.05% trifluoroacetic in 100% acetonitrile (v/v)
Total Flow:1 ml/min
Wavelength:220 nm

Time	Module	Command	Value
0.01	Pumps	Pump A B.Conc	5
25.00	Pumps	Pump A B.Conc	65
25.01	Pumps	Pump A B.Conc	95
27.00	Pumps	Pump A B.Conc	95
27.01	Pumps	Pump A B.Conc	5
35.00	Pumps	Pump A B.Conc	5
35.01	Controller	Stop	

<<Column Performance>>
<Detector A>
Column : Inertsil ODS-3 4.6 x 250 mm
Equipment: GK11010017

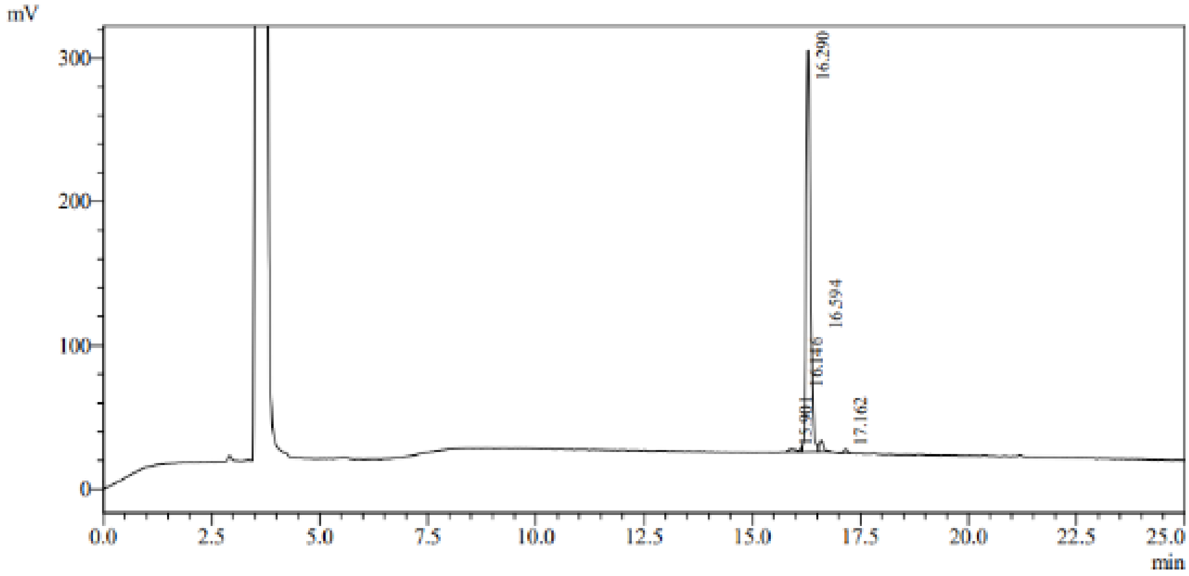

1 Detector A Channel 1 / 220nm

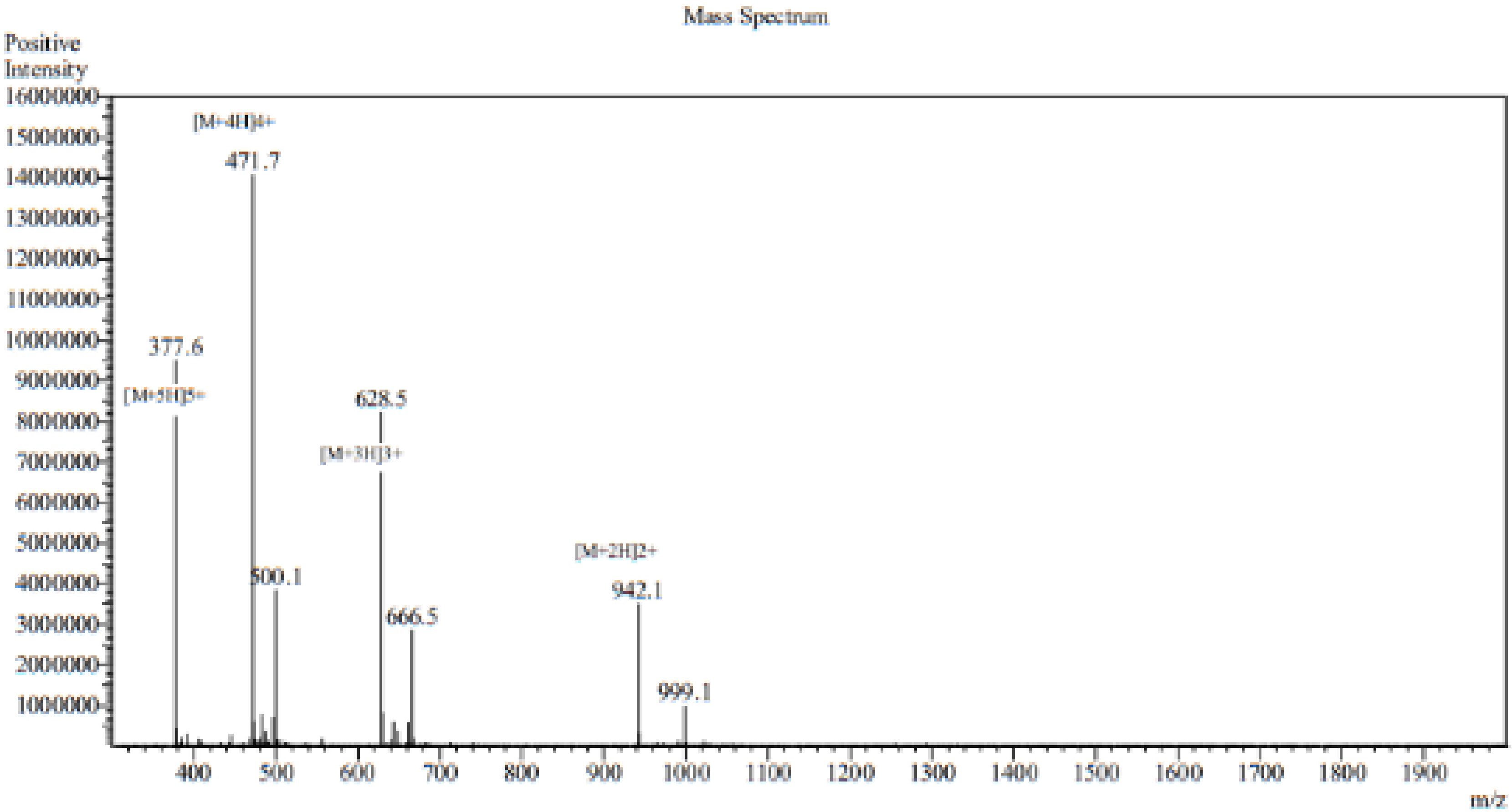

Sample Information	
Month-Day Processed :	02/11/22
Time Processed :	2:58:50 PM
Injection Volume :	0.4
Sample Name :	7L
Sample ID :	U929RHA240-13
Theoretical MW :	1882.28
Observed MW :	1882.8

Interface	:ESI
Nebulizing Gas Flow	:1.5L/min
CDL Temp	:250
Block Temp	:200

Equipment	: GK11010007
Interface Bias	: +4.5 kV
Drying Gas Flow	:5 L/min
T.Flow	:0.2 ml/min
B.conc	:50%H2O/50%MeOH

Sample Name :8L
Sample ID :U929RHA240-15
Time Processed :11:19:05
Month-Day-Year Processed :02/15/2022

Pump A : 0.065% trifluoroacetic in 100% water (v/v)
Pump B : 0.05% trifluoroacetic in 100% acetonitrile (v/v)
Total Flow:1 ml/min
Wavelength:220 nm

Time	Module	Command	Value
0.01	Pumps	Pump A B.Conc	5
25.00	Pumps	Pump A B.Conc	65
25.01	Pumps	Pump A B.Conc	95
27.00	Pumps	Pump A B.Conc	95
27.01	Pumps	Pump A B.Conc	5
35.00	Pumps	Pump A B.Conc	5
40.01	Controller	Stop	

<<Column Performance>>
<Detector A>
Column : Inertsil ODS-3 4.6 x 250 mm

Chromatogram

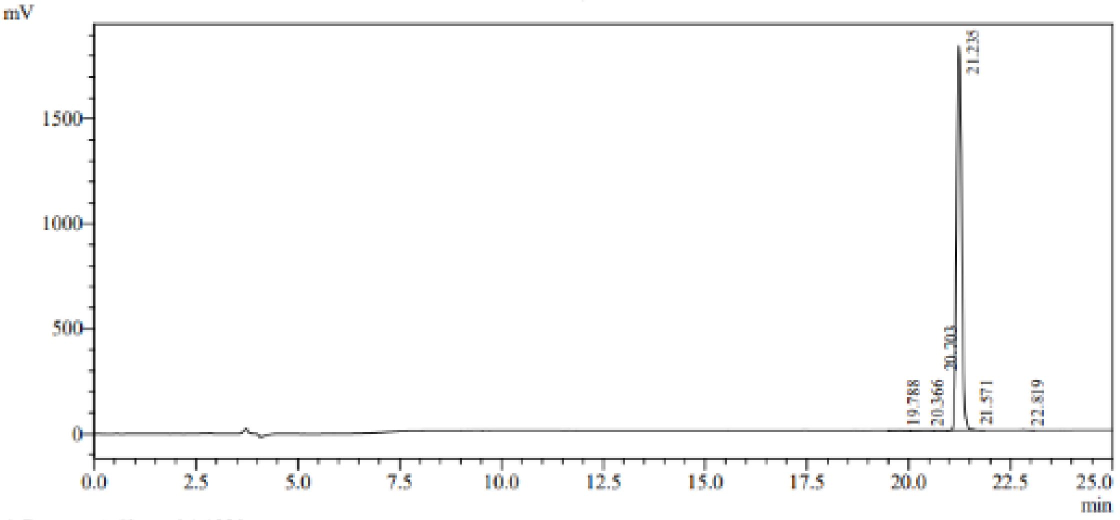

1 Detector A Channel 1 / 220nm

Mass Spectrum

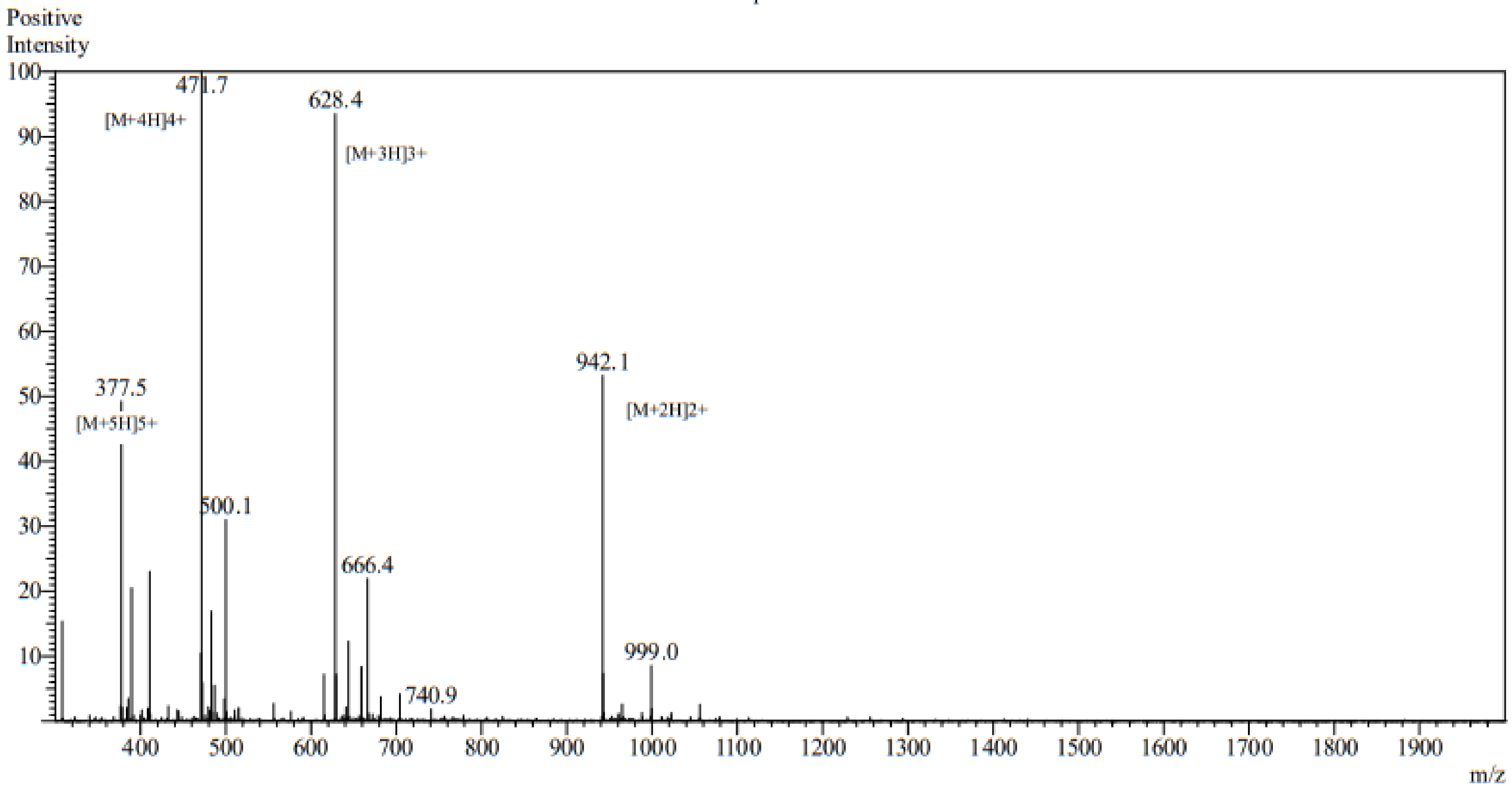

Sample Information	
Month-Day Processed :	02/15/22
Time Processed :	15:02:52
Injection Volume :	0.4
Sample Name :	8L
Sample ID :	U929RHA240-15
Theoretical MW :	1882.28
Observed MW :	1882.8

Interface	:ESI
Nebulizing Gas Flow	:1.5L/min
CDL Temp	:250
Block Temp	:200

Equipment	: ZJ21010035
Interface Bias	: +4.5 kV
Drying Gas Flow	:5 L/min
T.Flow	:0.2 ml/min
B.conc	:50%H2O/50%MeOH

References

1. Carias, L.L.; Rudin, S.D.; Donskey, C.J.; Rice, L.B. Genetic Linkage and Cotransfer of a Novel, VanB-Containing Transposon (Tn5382) and a Low-Affinity Penicillin-Binding Protein 5 Gene in a Clinical Vancomycin-Resistant Enterococcus Faecium Isolate. *J Bacteriol* **1998**, *180*, 4426–4434, doi:10.1128/JB.180.17.4426-4434.1998.

2. Donabedian, S.M.; Chow, J.W.; Boyce, J.M.; McCabe, R.E.; Markowitz, S.M.; Coudron, P.E.; Kuritza, A.; Pierson, C.L.; Zervos, M.J. Molecular Typing of Ampicillin-Resistant, Non-Beta-Lactamase-Producing Enterococcus Faecium Isolates from Diverse Geographic Areas. *J Clin Microbiol* **1992**, *30*, 2757–2761, doi:10.1128/jcm.30.11.2757-2761.1992.

3. Thorisdottir, A.S.; Carias, L.L.; Marshall, S.H.; Green, M.; Zervos, M.J.; Giorgio, C.; Mermel, L.A.; Boyce, J.M.; Medeiros, A.A.; Fraimow, H. IS6770, an Enterococcal Insertion-like Sequence Useful for Determining the Clonal Relationship of Clinical Enterococcal Isolates. *J Infect Dis* **1994**, *170*, 1539–1548, doi:10.1093/infdis/170.6.1539.

4. Garsin, D.A.; Sifri, C.D.; Mylonakis, E.; Qin, X.; Singh, K. v; Murray, B.E.; Calderwood, S.B.; Ausubel, F.M. A Simple Model Host for Identifying Gram-Positive Virulence Factors. *Proc Natl Acad Sci U S A* **2001**, *98*, 10892–10897, doi:10.1073/pnas.191378698.

5. Sahm, D.F.; Kissinger, J.; Gilmore, M.S.; Murray, P.R.; Mulder, R.; Solliday, J.; Clarke, B. In Vitro Susceptibility Studies of Vancomycin-Resistant Enterococcus Faecalis. *Antimicrob Agents Chemother* **1989**, *33*, 1588–1591, doi:10.1128/AAC.33.9.1588.

6. Dunny, G.M.; Brown, B.L.; Clewell, D.B. Induced Cell Aggregation and Mating in Streptococcus Faecalis: Evidence for a Bacterial Sex Pheromone. *Proc Natl Acad Sci U S A* **1978**, *75*, 3479–3483, doi:10.1073/pnas.75.7.3479.

7. Huycke, M.M.; Spiegel, C.A.; Gilmore, M.S. Bacteremia Caused by Hemolytic, High-Level Gentamicin-Resistant Enterococcus Faecalis. *Antimicrob Agents Chemother* **1991**, *35*, 1626–1634, doi:10.1128/AAC.35.8.1626.

8. Baba, T.; Takeuchi, F.; Kuroda, M.; Yuzawa, H.; Aoki, K.; Oguchi, A.; Nagai, Y.; Iwama, N.; Asano, K.; Naimi, T.; et al. Genome and Virulence Determinants of High Virulence Community-Acquired MRSA. *Lancet* **2002**, *359*, 1819–1827, doi:10.1016/s0140-6736(02)08713-5.

9. Vankerckhoven, V.; van Autgaerden, T.; Vael, C.; Lammens, C.; Chapelle, S.; Rossi, R.; Jabes, D.; Goossens, H. Development of a Multiplex PCR for the Detection of Asa1, GelE, CylA, Esp, and Hyl Genes in Enterococci and Survey for Virulence Determinants among European Hospital Isolates of Enterococcus Faecium. *J Clin Microbiol* **2004**, *42*, 4473–4479, doi:10.1128/JCM.42.10.4473-4479.2004.

10. Duprè, I.; Zanetti, S.; Schito, A.M.; Fadda, G.; Sechi, L.A. Incidence of Virulence Determinants in Clinical Enterococcus Faecium and Enterococcus Faecalis Isolates

Collected in Sardinia (Italy). *J Med Microbiol* **2003**, *52*, 491–498, doi:10.1099/jmm.0.05038-0.

11. Hashem, Y.A.; Amin, H.M.; Essam, T.M.; Yassin, A.S.; Aziz, R.K. Biofilm Formation in Enterococci: Genotype-Phenotype Correlations and Inhibition by Vancomycin. *Sci Rep* **2017**, *7*, 5733, doi:10.1038/s41598-017-05901-0.

12. Cui, P.; Feng, L.; Zhang, L.; He, J.; An, T.; Fu, X.; Li, C.; Zhao, X.; Zhai, Y.; Li, H.; et al. Antimicrobial Resistance, Virulence Genes, and Biofilm Formation Capacity Among Enterococcus Species From Yaks in Aba Tibetan Autonomous Prefecture, China. *Front Microbiol* **2020**, *11*, 1250, doi:10.3389/fmicb.2020.01250.
